# Roles of the Mesenchymal Stromal/Stem Cell Marker Meflin/Islr in Cancer Fibrosis

**DOI:** 10.3389/fcell.2021.749924

**Published:** 2021-10-05

**Authors:** Masahide Takahashi, Hiroki Kobayashi, Yasuyuki Mizutani, Akitoshi Hara, Tadashi Iida, Yuki Miyai, Naoya Asai, Atsushi Enomoto

**Affiliations:** ^1^International Center for Cell and Gene Therapy, Fujita Health University, Toyoake, Japan; ^2^Department of Pathology, Nagoya University Graduate School of Medicine, Nagoya, Japan; ^3^Department of Gastroenterology and Hepatology, Nagoya University Graduate School of Medicine, Nagoya, Japan; ^4^Department of Pathology, Fujita Health University, Toyoake, Japan

**Keywords:** Meflin, Islr, cancer-associated fibroblast, mesenchymal stromal/stem cell, tumour microenvironment, fibrosis

## Abstract

Fibroblasts synthesise the extracellular matrix (ECM) such as collagen and elastin, the excessive accumulation of which can lead to fibrosis and organ dysfunction under pathological conditions. Cancer-associated fibroblasts (CAFs) are major constituents of the tumour microenvironment (TME) that accompany the desmoplastic reaction responsible for anti-cancer treatment resistance. Thus, it is important to dissect the roles of CAFs in the TME to develop new therapeutic strategies for refractory cancers. Recent progress in the studies of CAF biology suggests that the functions of CAFs are complicated and that they are composed of functionally distinct populations, including cancer-promoting CAFs (pCAFs) and cancer-restraining CAFs (rCAFs). We recently identified a new cell surface marker for rCAFs in pancreatic and colon cancers, designated as Meflin (mesenchymal stromal cell- and fibroblast-expressing Linx paralogue)/Islr (immunoglobulin super family containing leucine-rich repeat). Based on the distribution of Meflin/Islr-positive cells, we also considered it a specific candidate marker for mesenchymal stroma/stem cells. Meflin/Islr-positive CAFs have been shown to suppress cancer progression by being involved in regulating collagen structures and BMP signalling in the TME. This review describes the function of Meflin/Islr in cancer fibrosis as well as in cardiac and lung fibrosis and its potential in the development of new cancer therapeutics.

## Introduction

Cancer consists of various cells, including cancer cells, fibroblasts, immune cells, and vessel components, and the tissue surrounding cancer cells is called the tumour microenvironment (TME) (Junttila and de Sauvage, 2013; Bejarano et al., 2021). In addition to these cells, the extracellular matrix (ECM) secreted by fibroblasts is its major constituent that can lead to the desmoplastic reaction that is conspicuous in aggressive and refractory cancers. Thus, much attention has been paid to not only the biological properties of cancer cells themselves but also the interaction between cancer cells and stromal cells to better understand the mechanisms of cancer progression (Chen and Song, 2019).

Cancer-associated fibroblasts (CAFs) are key components of TME that secrete a variety of extracellular matrices, including collagen and fibronectin, and promote fibrosis (Kalluri, 2016; Kobayashi et al., 2019; Miyai et al., 2020). Remarkable fibrosis is usually observed, especially in highly malignant cancers. For example, pancreatic ductal adenocarcinoma (PDAC), with a 5-year overall survival rate of approximately 10%, is characterised by a prominent fibrotic stromal reaction. CAFs are also known to produce secretory signalling molecules such as growth factors, cytokines, and chemokines, which are involved in the growth and progression of cancer cells, as well as the regulation of immune function and angiogenesis in the TME (Kalluri, 2016; Kobayashi et al., 2019).

It has been elucidated that the characteristics of CAFs are closely related to their tumour-promoting roles (Olumi et al., 1999; Orimo et al., 2005; Quante et al., 2011). However, interestingly, recent progress in TME studies showed that CAFs are heterogeneous populations, including cancer-promoting CAFs (pCAFs), cancer-restraining CAFs (rCAFs), and neutral CAFs (nCAFs), based on their functions (Kobayashi et al., 2019). Indeed, the complexity of CAF functions is demonstrated by data from both mouse models and clinical analyses. Several studies have also demonstrated the presence of certain CAF populations that suppress tumour growth (Zhang et al., 2013; Özdemir et al., 2014; Rhim et al., 2014; Shin et al., 2014; Maris et al., 2015; Pallangyo et al., 2015; Gerling et al., 2016).

Recently, our group found a new specific marker for rCAF, Meflin/Islr, in PDAC and colorectal cancer (CRC) (Mizutani et al., 2019; Kobayashi et al., 2021). Meflin/Islr is a glycosylphosphatidylinositol-anchored protein that was originally identified as a marker for mesenchymal stromal/stem cells (MSCs) (Maeda et al., 2016). In normal tissues, Meflin/Islr was detected in stromal cells distributed throughout the bone marrow (BM) and perivascular cells in multiple organs, including pancreatic stellate cells, which are known to be a source of CAFs in PDAC. Our study suggests that Meflin/Islr is necessary for maintaining the undifferentiated state of MSCs because its overexpression suppresses MSC differentiation. Consistently, its expression is markedly decreased during differentiation into osteoblasts, chondrocytes, adipocytes, and myofibroblasts in culture (Maeda et al., 2016). In contrast, Meflin/Islr-positive fibroblasts proliferate in the cancer stroma (Mizutani et al., 2019; Kobayashi et al., 2021) and in cardiac and lung fibrosis (Hara et al., 2019; Nakahara et al., 2021) under pathological conditions. In this review, we described and discussed the functions of Meflin/Islr in fibroblasts and their potential as targets for fibrotic diseases.

## Identification of Meflin/Islr as a Candidate Marker for Mesenchymal Stromal/Stem Cells

Mesenchymal stromal/stem cells are attracting attention in the field of regenerative medicine and are expected to be applied for medical use. MSCs are thought to have both self-renewal and multi-lineage differentiation abilities and are distributed in various tissues (Caplan, 1991; Pittenger et al., 1999). In culture, they can be induced to differentiate into osteoblasts, chondroblasts, adipocytes, fibroblasts, skeletal muscle cells, and neuronal cells (Beresford et al., 1992; Wakitani et al., 1995; Pittenger et al., 1999; Woodbury et al., 2000). However, due to the lack of MSC-specific markers, their distribution and characteristics have not been fully elucidated.

Mesenchymal stromal/stem cells in culture are defined by the expression of positive (CD73, CD90, and CD105) and negative (CD14, CD19, CD34, CD45, and HLA-DR) cell surface markers (Dominici et al., 2006). Other markers are also used for MSC isolation, including platelet-derived growth factor α (PDGFRα), Sca-1, Stro-1, CD106, CD146, and CD271 (Nombela-Arrieta et al., 2011; Andrzejewska et al., 2019). Moreover, a lineage-tracing approach using transgenic or knock-in mice revealed that MSCs positive for leptin receptor, GLI1 family zinc finger 1 (Gli1), paired related homeobox 1, chemokine (C-X-C motif) ligand 12, stem cell factor, and Gremlin 1 (Grem1) can produce differentiated osteocytes, chondrocytes, adipocytes, and fibroblasts (Omatsu et al., 2010; Greenbaum et al., 2013; Zhou et al., 2014; Kramann et al., 2015; Worthley et al., 2015). However, these markers are expressed not only by MSCs but also by other types of differentiated cells.

**FIGURE 1:**
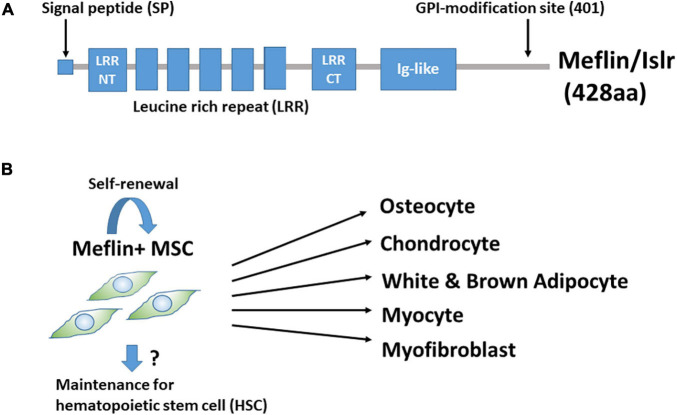
**(A)** Structure of Meflin/Islr. It is a glycosylphosphatidylinositol-anchored membrane protein with five leucine-rich repeats (LRRs) flanked by cysteine-rich N- and C-terminal domains and an immunoglobulin (Ig)-like domain. **(B)** Meflin/Islr is an MSC marker. Meflin is highly expressed in undifferentiated MSCs, whereas its expression is downregulated upon MSC differentiation. A lineage trace experiment showed the possibility that Meflin^+^ MSCs undergo self-renewal, although this has not yet been explicitly proven. Meflin^+^ MSCs may also be involved in the maintenance of heamatopoietic stem cells in the bone marrow.

We found that Meflin/Islr, a glycosylphosphatidylinositol-anchored cell surface protein, is expressed in cultured BM-derived MSCs and fibroblasts, but not in other types of cells, including epithelial, endothelial, smooth muscle, and neuronal cells (Figure 1A; Maeda et al., 2016). BM-MSCs were originally identified as colony-forming unit-fibroblasts in cultured BM cells (Nombela-Arrieta et al., 2011; Bianco, 2014; Andrzejewska et al., 2019). Meflin was most abundantly expressed in CD45^–^Ter119^–^PDGFα^+^Sca-1^+^ cells in the BM, which is highly enriched for colony-forming unit-fibroblast activity (Maeda et al., 2016). Interestingly, *in situ* hybridisation analyses revealed that Meflin-positive cells were sparsely detected in the BM and perivascular regions of various organs. It was intriguing that many of them in the BM were located adjacent to the perisinusoidal regions and their frequency was estimated to be less than a few percent of all nucleated cells. Meflin expression was also not detected in any heamatopoietic lineage in the BM. Meflin is also expressed in some of the perivascular cells across various organs, including the skin, heart, pancreas, intestine, skeletal muscle, adipose tissue, and brain, which comprise MSCs. Altogether, our findings suggest that Meflin represents a potential specific cell surface marker of MSCs that are distributed throughout the body. In addition, we showed that Meflin expression was markedly decreased or became negative when BM-MSCs were differentiated into osteoblasts, chondroblasts, adipocytes, and myofibroblasts *in vitro* (Figure 1B; Maeda et al., 2016). This finding implies that Meflin may be necessary for the maintenance of undifferentiated MSCs.

As mentioned above, it has been shown that cells expressing the leptin receptor, paired related homeobox 1, chemokine (C-X-C motif) ligand 12, Gli1, or Grem1 in the bone and BM give rise to differentiated osteocytes, chondrocytes, adipocytes, and fibroblasts (Omatsu et al., 2010; Greenbaum et al., 2013; Zhou et al., 2014; Kramann et al., 2015; Worthley et al., 2015). However, information regarding the differentiation capacity of these cells outside the bone is limited. Thus, we performed lineage-tracing experiments to study the fate of Meflin-positive cells throughout the body. We used a knock-in mouse line expressing constitutive or tamoxifen (TAM)-inducible Cre recombinase (CreERT2) under the control of the Meflin promoter. A mouse line was crossed with a Rosa26-LoxP-stop-LoxP (LSL)-tdTomato gene to detect Meflin-lineage cells by tdTomato expression. Our data clearly demonstrated that Meflin-positive cells in various mesenchymal tissues can give rise to mature osteocytes, chondrocytes, adipocytes, and skeletal myocytes in the postnatal and adult stages (Hara et al., 2021). Another previous study showed the differentiation of Meflin-positive cells into mature white adipocytes and beige-like adipocytes under physiological and cold-stress conditions, respectively, using mice carrying a transgene in which the Meflin promoter drives the expression of the TAM-inducible Cre recombinase (Kuwano et al., 2021).

When TAM was administered at postnatal day 1 (P1), P2, and P3, we found that some differentiated Meflin lineage cells (e.g., adipocytes or chondrocytes) were detected in groups or clusters at P21 and P49, suggesting that they may have originated from the same MSCs or progenitors (Hara et al., 2021). In addition, most tdTomato^+^ Meflin-lineage cells in the BM were also positive for PDGFRα(a marker of the most primitive MSCs in the BM) at P21 and P49. Thus, Meflin-positive MSCs are likely to give rise to both PDGFRα-positive undifferentiated stromal cells and mature lineage cells, maintaining both self-renewal capacity and differentiation potential (Figure 1B). Given the view that MSCs in the BM provide niches for hematopoietic stem cells (HSCs) (Wei and Frenette, 2018), Meflin-positive cells may also be involved in the maintenance of HSCs. Moreover, it has been reported that Meflin is expressed by skeletal muscle stem cells and plays a crucial role in skeletal muscle regeneration *via* the canonical Wnt signalling pathway (Zhang et al., 2018).

## Role of Meflin-Positive Cancer-Associated Fibroblast in Pancreatic Carcinogenesis

Many studies have demonstrated that CAFs increase cancer malignancy and therapeutic resistance (Ishii et al., 2016; Kalluri, 2016; Chen and Song, 2019). Several proteins, such as αSMA, fibroblast activation protein (FAP), fibroblast-specific protein 1, and podoplanin are known as established CAF markers (Kobayashi et al., 2019), and CAF infiltration is correlated with poor outcomes in cancer patients (Kawase et al., 2008; Fujita et al., 2010; Valach et al., 2012; Yamashita et al., 2012; Sinn et al., 2014; Underwood et al., 2015; Alcaraz et al., 2019). Thus, clinical trials targeting CAFs are emerging as new cancer therapeutics. However, recent observations have revealed that the functions of individual CAF are not necessarily the same in the cancer stroma. Genetic depletion of αSMA^+^-CAFs or blocking of sonic hedgehog (Shh) signalling, which is responsible for desmoplastic reaction in PDAC, resulted in the progression of PDAC developed in LSL-KrasG12D; LSLTrp53R172H; PDX-1-Cre (KPC) mice, proving that rCAF subpopulations are present in the stroma of PDAC (Gore and Korc, 2014; Lee et al., 2014; Rhim et al., 2014; Özdemir et al., 2014).

While analysing Meflin expression in the whole body, we found that it is detected in pancreatic stellate cells (PSCs) that express desmin. Because PSCs are known to be a source of CAFs in PDAC, this finding prompted us to study the roles of Meflin-positive CAFs in the TME of PDAC.

**FIGURE 2:**
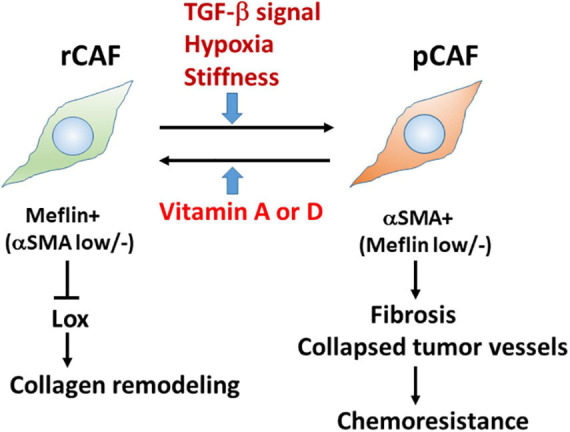
Meflin is a new marker for restraining cancer-associated fibroblast (rCAFs) in pancreatic cancer. Recent evidence showed that Meflin^+^ rCAFs, which suppress the activity of Lox and Lox-mediated collagen remodelling, differentiate into αSMA^+^ pCAFs that are weakly positive or negative for Meflin and promote tissue fibrosis during cancer progression. TGF-β signalling, hypoxia, and stiffness are major factors that induce the phenotypic conversion of CAFs. Conversely, it has been hypothesised that vitamins A and D could reprogram pCAFs to rCAFs.

Our *in situ* hybridisation study revealed that Meflin-positive cells were sparsely detected in the perivascular, periductal, and periacinar areas of the pancreas, including desmin-positive PSCs. Consistent with the fact that gene expression in PSC is regulated by vitamin D (Sherman et al., 2014), Meflin-positive cells express vitamin D receptor, and Meflin expression itself is upregulated by treatment with calcipotriol, which is a vitamin D analogue (Figure 2; Mizutani et al., 2019).

In PDAC, PSCs are suspected to be CAFs that play a crucial role in their progression. In fact, we found Meflin-positive fibroblasts in the stroma of PDAC, although their number and density varied significantly depending on the tumour. A major fraction of PDGFα-positive cells and Gli1 (a transcription factor involved in the sonic hedgehog pathway)-positive cells were also positive for Meflin, whereas strong Meflin-positive cells were negative or weakly positive for αSMA. In addition, Meflin was expressed in approximately 40% of FAP-positive CAFs, suggesting that Meflin defines a subtype of CAFs, that is, αSMA^–/low^ FAP^±^ PDGFRα^+^ Gli1^+^ in PDAC (Mizutani et al., 2019).

We investigated the impact of Meflin expression in the stroma on the outcomes of patients with PDAC. We divided the patients into Meflin-high (>20% Meflin-positive stromal cells) and Meflin-low (<20% Meflin-positive stromal cells) groups. Interestingly, the Meflin-high group exhibited significantly better prognosis than the Meflin-low group, suggesting a unique feature of Meflin-high fibroblasts that may represent rCAFs (Mizutani et al., 2019). We also evaluated the significance of Meflin expression in the progression of PDAC in KPC mice by crossing them with Meflin-KO mice. Meflin knocked-out (KO) KPC mice developed larger and more proliferative tumours than the wild-type (WT) KPC mice, further supporting the suppressive role of Meflin in tumour development.

Interestingly, PDAC developed in Meflin-KO mice showed a poorly differentiated type compared with that in WT mice; the former contained more αSMA^+^ CAFs and more collapsed tumour vessels than the latter (Figure 2; Mizutani et al., 2019). In contrast, the expression of FAP, a pCAF marker, was not significantly different between the WT and Meflin-KO KPC mice. Thus, the levels of Meflin expression in CAFs seem to influence αSMA expression and play a role in tumour differentiation through the interplay between cancer cells and CAFs. Ageing, hypoxia, and TGF-β signalling induce the downregulation of Meflin expression, whereas vitamin D induces its upregulation (Figure 2; Hara et al., 2019; Mizutani et al., 2019). Although the mechanisms underlying the downregulation of Meflin expression in CAFs remain unknown, our data suggest that the balance of Meflin-positive CAFs and αSMA-positive CAFs in the TME could affect tumour progression and differentiation (Miyai et al., 2020).

To trace Meflin-positive cells in transplanted PDAC tumours, we used a knock-in mouse line in which the expression of CreERT2 was driven by the Meflin promoter. This mouse line was crossed with Rosa26-LSL-tdTomato mice, which allowed us to trace Meflin-lineage cells in the stroma of transplanted PDAC tumours by TAM administration. *In situ* hybridisation analysis revealed that the number of tdTomato^+^ Meflin-lineage cells that express αSMA significantly increased as the tumours grew after transplantation. Although Meflin expression can suppress the expression of αSMA *in vitro*, lineage-tracing experiments have also suggested that Meflin^+^ cells can convert to αSMA^+^ CAFs during tumour progression (Mizutani et al., 2019). Thus, it seems plausible that the stromagenic switch from rCAF to pCAF occurs in the PDAC stroma (Figure 2).

It is important to determine the biological function of Meflin in the TME. As mentioned above, histochemical analysis showed an increase in collapsed tumour vessels in the Meflin-KO mice, suggesting the involvement of Meflin in stromal collagen structural remodelling. Consistently, the second-harmonic generation microscopic observations demonstrated that the stroma of Meflin-KO KPC tumours exhibited straighter and wider collagen structures than those of WT tumours (Mizutani et al., 2019). The alteration of collagen configuration in Meflin-KO mice also appears to correlate with the increase in stiffness in the cancer stroma. Our recent study identified lysyl oxidase (Lox) with the activity of ECM cross-linking as a Meflin-binding protein by mass spectrometry (Iida et al., 2021). Altogether with the findings of second-harmonic generation microscopy, this suggests that Meflin may function as a suppressor of Lox in the cancer stroma, leading to changes in collagen structures (Figure 2).

## Role of Meflin-Positive Cancer-Associated Fibroblast in Colorectal Carcinogenesis

Bone morphogenetic protein (BMP) signalling regulates cell proliferation and tumour development, including CRC (Bach et al., 2018). In the normal colon, the epithelial stem cell niche is maintained by low BMP and high Wnt at the crypt base, whereas epithelial cell differentiation is induced by high BMP and low Wnt towards the luminal surface (Jung et al., 2017). The BMP inhibitors Grem1 and Noggin are secreted by fibroblasts at the crypt base, and they generate the BMP gradient necessary for the intestinal epithelial stem cell niche (Worthley et al., 2015; Jung et al., 2017; McCarthy et al., 2020). In addition, BMP signalling inactivation contributes to CRC predisposition and progression, indicating its inhibitory role in colorectal carcinogenesis (He et al., 2004; Qi et al., 2017; Bach et al., 2018).

**FIGURE 3:**
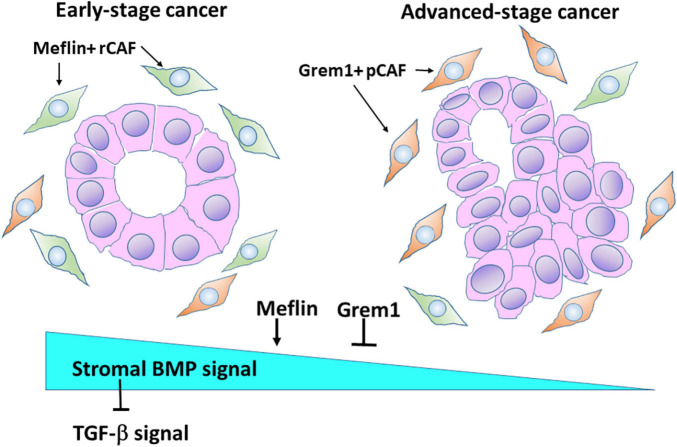
Meflin and Grem1 expression in CAFs regulates stromal BMP signalling involved in tumour progression. The collective evidence from several studies shows that Meflin^+^ rCAFs proliferate in early-stage cancer, in which Meflin may augment stromal BMP signalling that counteracts with TGF-β signalling. In advanced-stage cancer, Grem1^+^ pCAFs may be dominant and suppress BMP signalling to promote cancer progression.

We recently identified Grem1 and Meflin/Islr as CAF-specific genes involved in BMP signalling in the CRC stroma (Kobayashi et al., 2021). Interestingly, these two genes were expressed in distinct subpopulations of CRC CAFs (Figure 3). Grem1 and Meflin are significantly upregulated in human CRC CAFs compared to normal colon fibroblasts, and their highest expression was observed in CAFs of a stroma-rich molecular subtype of CRC [consensus molecular subtype 4 (CMS4)]. Survival analyses indicated that high Grem1 and Meflin expressions were associated with poorer survival and improved survival, respectively. In addition, Grem1 expression levels in CRC CAFs were inversely correlated with Meflin expression, and Grem1 was predominantly expressed in myofibroblasts with αSMA expression.

Another interesting finding is that in the normal colon, Grem1 expression was detected in fibroblastic cells near the base of the colon crypts, whereas Meflin-positive fibroblasts were observed near the middle of them, indicating that the distribution of Grem1-positive cells and Meflin-positive cells is topographically distinct. In addition, Grem1 expression was detected in Foxl1-positive telocytes, which are stromal cells that provide key intestinal stem cell niche signalling molecules. In contrast, Meflin positive fibroblasts exhibited lower Foxl1 positivity (Kobayashi et al., 2021). In agreement with these findings, Grem1/Meflin-double positive cells were very few in the stroma of CRC compared with the number of single-positive cells, implying distinct roles of both proteins in CRC CAFs.

Our studies clearly demonstrated the opposing roles of Grem1 and Meflin in BMP signalling. Conditioned medium from Meflin-overexpressing cells augmented signalling (Smad1/5 phosphorylation) and gene expression (*Id1* and *Id2* expression) mediated by BMP2 and BMP7. In contrast, conditioned medium from Grem1-overexpressing cells decreased BMP-mediated signalling and gene expression. Thus, the expression levels of Grem1 and Meflin seem to fine-tune BMP signalling in the CRC stroma, which influences cancer progression and differentiation. In addition, BMP signalling could antagonise TGF-β signalling, which promotes a desmoplastic reaction in the TME of CRC (Figure 3).

Liver metastasis is a major cause of CRC deaths. Using a mouse model of CRC liver metastasis, we evaluated the effect of adeno-associated virus 8 (AAV8, a serotype with tropism for murine hepatocytes) carrying the *Islr* gene on the growth of metastatic tumours. AAV8-*Islr* was infected into hepatocytes *via* mouse tail vein, and 2 weeks later, *Apc^Δ^ ^/Δ^ Trp53^Δ^ ^/Δ^* mouse CRC organoids (referred to as *AP* tumouroids) were injected into the portal vein to generate CRC liver metastasis. The liver-directed delivery of *Islr* enhanced BMP signalling in liver metastasis, repressed tumour growth, and prolonged mouse survival, indicating the importance of BMP signalling in metastatic regions of CRC, similar to its original site (Kobayashi et al., 2021). This result also suggests that AAV-mediated therapy could be promising for the treatment of liver metastasis in CRC.

## Roles of Meflin in Heart and Lung Fibrosis

The excessive deposition of ECM components secreted from fibroblasts can lead to the disruption of tissue architecture and organ dysfunction (Kramann et al., 2013; Gourdie et al., 2016; Tallquist and Molkentin, 2017). Understanding the molecular basis of fibrosis in each organ is essential for therapeutic interventions (El Agha et al., 2017). As observed in CAFs, recent studies revealed that fibroblast heterogeneity can also be recognised in tissues and fibrotic diseases (Lynch and Watt, 2018; Henderson et al., 2020). Each tissue, including the heart, lung, gastrointestinal tract, and muscle, appears to contain fibroblasts with specific functions.

Meflin-positive fibroblastic cells are sparsely detected in the heart and lung. Similar to the cancer stroma, we found the proliferation of Meflin-positive cells and their lineage cells in the hearts after acute myocardial infarction (MI) and pressure-overload heart failure mouse models as well as in fibrotic foci of pulmonary fibrosis in humans and mice (Hara et al., 2019; Nakahara et al., 2021).

In MI hearts, Meflin-positive fibroblasts proliferated in the necrotic zone and the border area between necrotic and viable tissues in the reparative phase. In the fibrotic phase after the MI induction, Meflin-lineage cells express myofibrotic markers such as αSMA and vimentin, indicating that they give rise to myofibroblasts, which is consistent with the fact that Meflin-positive cells are converted to αSMA-positive cells in the cancer stroma. Interestingly, when the MI model was subjected to Meflin-KO mice, they exhibited shorter survival due to cardiac rupture than WT mice (Hara et al., 2019). Meflin-KO hearts after MI induction expressed more αSMA and interleukin-6, which mediate the acute response to tissue injury. Moreover, using the transverse aortic constriction mouse model, we found that Meflin-KO mice exhibit poor prognosis and advanced fibrosis compared with WT mice. The measurement of the elasticity of the heart surface by atomic force microscopy revealed that Meflin-KO mice developed stiffer failing hearts than WT mice. These findings suggest that Meflin-KO mice could be defective in the repair of injured heart or the control of inflammation after MI and chronic heart failure. Stiffer failing hearts in Meflin-KO mice may be caused by altered collagen structures and decreased BMP signalling.

The prognosis of idiopathic pulmonary fibrosis (IPF) remains poor despite recent therapeutic advances (du Bois, 2012; Lederer and Martinez, 2018). The pathological characteristics of IPF include fibroblastic foci that may progress to dense fibrosis (King et al., 2001; Nicholson et al., 2002). It has also been demonstrated that fibroblasts isolated from IPF are heterogeneous and have properties different from those of normal lungs (Thannickal et al., 2004; Wynn, 2011). Our recent studies revealed that in IPF, more than 70% of fibroblasts were positive for Meflin in the lesions of fibroblastic foci, approximately 50% of which were negative for αSMA. In contrast, more than 70% of fibroblasts were αSMA-positive myofibroblasts in dense fibrotic lesions, and more than 95% of myofibroblasts were negative for Meflin (Nakahara et al., 2021). This finding indicates the heterogeneity of fibroblasts at different stages in IPF and that αSMA-positive fibroblasts become dominant in the process of formation of dense fibrotic lesions from fibroblastic foci.

To further analyse the role of Meflin in lung fibrosis, a bleomycin (BLM)-induced lung fibrosis mouse model was used. Western blot analyses revealed that Meflin expression increased in a time-dependent manner in BLM-treated lungs with increasing numbers of Meflin-positive cells in the fibrotic lesions. Lungs from BLM-treated Meflin-KO mice exhibited more severe pulmonary fibrosis than those from BLM-treated WT mice. In addition, the expression of fibronectin and αSMA, collagen content, and smad2 activation were significantly higher in the BLM-KO group than in the BLM-WT group, suggesting a protective role of Meflin in anti-fibrotic effects against lung fibrosis (Nakahara et al., 2021). The effects of Meflin on the pathogenesis of lung fibrosis and heart failure may also be mediated by enhancing BMP signalling and antagonising TGF-β signalling, as observed in cancer fibrosis.

## Conclusion

Meflin/Islr represents a new specific MSC marker whose positive cells are sparsely distributed, particularly in the perivascular region of various tissues in the whole body (Maeda et al., 2016). Meflin expression markedly decreased during differentiation into osteoblasts, chondrocytes, adipocytes, and muscle cells, suggesting that Meflin functions to maintain the undifferentiated state of MSCs. In agreement with this view, Meflin overexpression suppressed the expression of osteoblastic and chondrocyte differentiation markers, runt-related transcription factor 2 and sex-determining region Y-box 9, respectively (Maeda et al., 2016). In addition, Meflin-positive fibroblasts and CAFs proliferate in cardiac and lung fibrosis and in the cancer stroma, respectively, and suppress disease progression (Hara et al., 2019; Mizutani et al., 2019; Kobayashi et al., 2021; Nakahara et al., 2021).

Until recently, CAFs have been considered to induce cancer progression and chemoresistance (Olumi et al., 1999; Orimo et al., 2005; Jena et al., 2020; Jena and Mandal, 2021). However, studies using single-cell transcriptome and proteome analyses indicated the presence of functionally and molecularly heterogeneous CAFs, including pCAFs and rCAFs. Various CAF markers, including αSMA, FAP, Grem1, and fibroblast-specific protein 1, have been identified (Kobayashi et al., 2019), but rCAF-specific markers have not been well characterised. Interestingly, we found that Meflin represents a marker for rCAFs in the stroma of PDAC and CRC, the high percentages of which are associated with better patient outcomes (Mizutani et al., 2019; Kobayashi et al., 2021). Recent reports have shown that interleukin-1 and TGF-β, which may be derived from cancer cells, can induce interleukin-6^+^ CAFs and αSMA^+^ CAFs in PDAC, respectively (Öhlund et al., 2017; Biffi et al., 2019). Moreover, using a mouse model of breast cancer, four groups of CAFs, including vascular CAF, matrix CAF, cycling CAF, and developmental CAF, were classified (Bartoschek et al., 2018). These studies demonstrate the presence of distinct populations of CAFs, although the relationship between these subpopulations and Meflin-positive CAFs remains unclear. A more complete picture of CAF diversity in the cancer stroma should be elucidated by single-cell transcriptome and proteome analyses to better understand the biological significance of CAFs.

Meflin binds to BMP-7 and enhances BMP signalling in the CRC stroma, which counteracts the action of Grem1 and TGF-β(Figure 3; Kobayashi et al., 2021). High Meflin expression suppressed αSMA expression in CAFs, preventing excessive fibrosis in the TME. This also seems to be the case in cardiac and lung fibrosis, because Meflin expression in fibroblasts results in favourable outcomes. Numerous studies have demonstrated the pathological roles of TGF-β in the progression of organ fibrosis and cancer fibrosis (Chen and Song, 2019; Shi et al., 2020). Thus, the augmentation of BMP signalling by Melfin is highly relevant for the development of new anti-fibrotic therapeutic interventions.

In addition, our data suggest that Meflin suppresses the activity of Lox family proteins, resulting in a decrease in ECM cross-linking in the TME (Mizutani et al., 2019; Iida et al., 2021). If the cancer stroma becomes soft due to Meflin expression in CAFs, chemosensitivity would be improved. We showed by lineage-tracing experiments that rCAFs expressing Meflin can convert to pCAFs expressing αSMA during tumour progression. A challenging issue is whether pCAFs can be reprogrammed into rCAFs in the TME. It has been reported that the administration of calcipotriol, a vitamin D analogue, or all-*trans*-retinoic acid, a vitamin A derivative, could possibly reprogram activated PSCs to a more quiescent state (Figure 2), which sensitises PDAC to chemotherapy and inhibits PDAC progression. Given the plasticity of CAFs *in vivo*, combination therapies using vitamin A or D analogues and other anti-cancer drugs are promising and currently underway in clinical trials for cancer patients.

## Author Contributions

MT wrote the manuscript. HK, YaM, AH, TI, YuM, NA, and AE reviewed and revised the manuscript. All authors contributed to the article and approved the submitted version.

## Conflict of Interest

The authors declare that the research was conducted in the absence of any commercial or financial relationships that could be construed as a potential conflict of interest.

## Publisher’s Note

All claims expressed in this article are solely those of the authors and do not necessarily represent those of their affiliated organizations, or those of the publisher, the editors and the reviewers. Any product that may be evaluated in this article, or claim that may be made by its manufacturer, is not guaranteed or endorsed by the publisher.
